# Shifting chronic disease management from hospitals to primary care in Estonian health system: analysis of national panel data

**DOI:** 10.7189/jogh.06.020701

**Published:** 2016-12

**Authors:** Rifat Atun, Ipek Gurol–Urganci, Thomas Hone, Lisa Pell, Jonathan Stokes, Triin Habicht, Kaija Lukka, Elin Raaper, Jarno Habicht

**Affiliations:** 1 Harvard T.H. Chan School of Public Health, Boston, MA, USA; 2 Department of Health Services Research & Policy, London School of Hygiene & Tropical Medicine, London, UK; 3 Department of Primary Care and Public Health, Imperial College, London, UK; 4 The Centre for Global Child Health, The Hospital for Sick Children, Toronto, Canada; 5 NIHR Greater Manchester Primary Care Patient Safety Translational Research Centre, Manchester Academic Health Science Centre, University of Manchester, Manchester, UK; 6 Ministry of Social Affairs, Tallinn, Estonia; 7 Estonian Health Insurance Fund, Tallinn, Estonia; 8 WHO Country Office in Republic of Kyrgyzstan, World Health Organization

## Abstract

**Background:**

Following independence from the Soviet Union in 1991, Estonia introduced a national insurance system, consolidated the number of health care providers, and introduced family medicine centred primary health care (PHC) to strengthen the health system.

**Methods:**

Using routinely collected health billing records for 2005–2012, we examine health system utilisation for seven ambulatory care sensitive conditions (ACSCs) (asthma, chronic obstructive pulmonary disease [COPD], depression, Type 2 diabetes, heart failure, hypertension, and ischemic heart disease [IHD]), and by patient characteristics (gender, age, and number of co–morbidities). The data set contained 552 822 individuals. We use patient level data to test the significance of trends, and employ multivariate regression analysis to evaluate the probability of inpatient admission while controlling for patient characteristics, health system supply–side variables, and PHC use.

**Findings:**

Over the study period, utilisation of PHC increased, whilst inpatient admissions fell. Service mix in PHC changed with increases in phone, email, nurse, and follow–up (vs initial) consultations. Healthcare utilisation for diabetes, depression, IHD and hypertension shifted to PHC, whilst for COPD, heart failure and asthma utilisation in outpatient and inpatient settings increased. Multivariate regression indicates higher probability of inpatient admission for males, older patient and especially those with multimorbidity, but protective effect for PHC, with significantly lower hospital admission for those utilising PHC services.

**Interpretation:**

Our findings suggest health system reforms in Estonia have influenced the shift of ACSCs from secondary to primary care, with PHC having a protective effect in reducing hospital admissions.

Estonia, a Baltic state with a population of 1.3 million people [1], has introduced comprehensive health system reforms following independence from the Soviet Union in 1991. The reforms (**Box 1**), which were aimed at improving population health, providing financial risk protection to citizens, enhancing user satisfaction and ensuring financial sustainability, centred on strengthening primary health care (PHC), introduction of a national health insurance system, and consolidating the number of hospitals [2].

Box 1 **Key reforms and policy charges in the Estonian Health System 2005–2012**

**2006**: Quality bonus system implemented in PHC to promote disease prevention and management of selected chronic conditions [2]; Family nurse services also expanded.

**2008**: Nationwide e–health system (integrated electronic health records, e–prescriptions, digital imaging, and laboratory tests, allowing information exchange between clinical, research and managerial professionals, with data confidentiality governed by strict protection laws, whilst providing patients access and control of their records [3]) launched to improve efficiency by reducing paperwork and duplication.

**2009**: Adoption of the Primary Health Care Development Plan that emphasized comprehensive service provision in PHC, prevention, chronic disease management, and improvements to access and care continuity [3]; Health service tariffs reduced by 6% due to economic crisis; maximum waiting times for outpatient specialist visits extended from four to six weeks; outpatient specialist care given higher priority over inpatient care to contain expenditure [2].

**2010**: Amendment to decree on prescribing and dispensing drugs requiring pharmacies to provide patients with the cheapest generic drug; Expansion of the role of family nurses to provide consultations and counselling to key groups, including chronically ill, pregnant women and healthy neonates. School nurses provide all school health services and immunizations. Midwives can prescribe in certain cases and operate individual practices.

**2011**: Health service payment tariffs increased by 1% in general, and by 3% in PHC to encourage higher use of PHC and lower use of hospitals.

**2012**: Tariffs increased to pre–crisis level, with the differential between PHC and hospitals introduced in 2011 maintained; Centralization of planning and regulatory functions, with PHC management functions centralized from country governors to the National Health Board; Amendment to Health Insurance Act to strengthen the gatekeeping function of PHC by reducing the number of directly accessible specialties in outpatients; Age–adjusted capitation payment scheme strengthened to motivate family doctors to treat more patients with chronic conditions and improve management of chronic conditions.

Family medicine was established as a specialty in Estonia in 1993, and in 1997 all citizens were required to register with a family physician, who were established as independent providers or from 2008 employed by municipalities [4,5]. The family medicine model placed PHC at the centre of the health system to improve quality, gatekeeping and care–coordination [6,7], and to address inefficiencies inherited from the Soviet–style hospital–centred health system, by reducing excessive referral and inpatient admissions to hospitals [6].

Estonia transitioned to an insurance–based financing model in 1991 with the creation of regional sickness funds [6], followed by the establishment of the independent Estonian Health Insurance Fund (EHIF) in 2001 as the national agency responsible for purchasing health care and contracting with health care providers [3]. From its inception, the EHIF implemented a purchasing strategy that prioritised outpatient care over inpatient hospital care through contracting targets and by reallocating funding [3]. Other supply–side changes aimed at improving service quality and efficiency included introduction of clinical guidelines [8], and using new provider payment mechanisms (capitation and of pay–for–performance [P4P] in PHC, and diagnosis–related groups (DRGs) in hospitals).

Strong PHC is associated with more equitable and accessible health care, greater efficiency, reduced emergency care, and better health outcomes [9,10], though few national–level empirical studies exist[10]. Analysis of the Estonian health system reforms offers, therefore, the opportunity to assess the countywide effect of introducing family medicine centred PHC on health service utilisation, and specifically on the management of chronic conditions. Nationwide individual level data on health care utilisation has been collected by the EHIF since 2000/2001. Data on health system supply–side variables (including health care providers and professionals) and demographic and socio–economic variables allow us to investigate the nationwide effect of health system changes after controlling for other factors influencing health service utilisation.

We examine how the Estonian health system reforms have affected service utilization across 2005–2012 in family medicine clinics and outpatient departments, and admissions to hospital for selected ambulatory–care sensitive conditions (ACSCs); conditions which should be effectively managed in PHC [11,12]. In a health system with appropriate access to [13] and effective provision of [14] PHC, hospital admissions for ACSCs should largely be avoidable. Study of Estonia is timely, as PHC is critical for achieving universal health coverage (UHC) [15], for creating a patient–centred health systems and for efficient and effective management non–communicable diseases (NCDs) [16,17].

## METHODS

The study used data from the EHIF administrative data set, which contains patient level records of all PHC, outpatient and inpatient contacts. While the EHIF data has been collected since 2000/2001, data completeness was achieved in 2005 when quality assurance of the data set was standardised. Therefore, the study period is limited to 1 January 2005 to 31 December 2012.

In the period 2005 and 2012, the EHIF data set contains 35.6 million PHC records, 22.2 million outpatient records and 1.7 million inpatient records, covering 1.1–1.2 million patients per year and all health care utilisation episodes for Estonian citizens.

The EHIF uses an electronic invoicing system with controls that ensure all submitted invoices have appropriate patient data, diagnoses and other relevant information related to the contact with the health system. In addition, EHIF uses a retrospective data quality analytical reporting system to identify systematic outliers that are not possible to detect during invoicing. As providers are paid for the services they provide, there is unlikely to be under–reporting, and electronic fraud and quality checking mechanisms ensure data reporting and coding quality.

Seven ACSCs were selected for the analysis: asthma, chronic obstructive pulmonary disease (COPD), depression, Type 2 diabetes, heart failure, hypertension, and ischaemic heart disease (IHD). These ACSCs, which are well–established in the literature and have been used in earlier studies [14,18], account for a proportionally high disease burden in Estonia [19]. We included depression in our list of ACSCs–a relatively prevalent mental health condition that accounts for a high burden of illness and disability [16], with high levels of hospital admissions [20], and common in patients with multi–morbidity [21].

Records for all episodes of care for patients aged 15 years and older, with a primary diagnosis of the seven ACSCs were extracted from the EHIF database. In the database, diagnostic information is coded using the international classification of diseases, 10^th^ revision (ICD–10) [22]. All episodes of care with the following ICD 10 codes were eligible for inclusion: asthma (J45), COPD (J44.9), depression (F32), diabetes (E11), heart failure (I50), hypertension (I10, I11–I15) and IHD (I20 & I25).

For all episodes of care, the start and end date of the health care invoice, age, gender, the county of residence of the patient, and the primary diagnosis are recorded. For PHC episodes, details on the service provider (family physician or family nurse) and type of consultation (new vs follow–up, preventive, home visit, telephone or email consultation) are also available.

We obtained data on county level health system and demographic variables from the Statistics Estonia website [23] and the Health Statistics and Health Research Database [24]. These data included total population (by age group); the number of doctors and nurses (full–time equivalents (FTEs)) working in family doctors’ offices; number of hospitals and hospital beds; number of family doctor offices; employment rate (individuals aged 15–64 years); and mean yearly disposable income.

To explore changes in PHC provision, we investigated changes in the total number of doctors and nurses per population, as well as PHC practice patterns over time. We analysed PHC practice patterns for ACSCs by service provider (family doctor vs nurse), type (new episode, follow–up or preventative), and location (office, home, phone/email).

For analysis of national trends in health service utilisation, the total number of yearly health service contacts for all patients in each diagnosis group was aggregated for primary, outpatient, and inpatient care. Utilisation trends were summarized for each health condition using age–standardised service utilisation rates per 100 000 population. We also compared the proportional distribution of service use between different services for each health condition analysed.

For analyses using patient level data, a data set of patient–year observations was created. Unique patient identification numbers in EHIF allow for patients to be followed in time and across primary and secondary care levels. Therefore, the resulting patient–year data was a longitudinal (panel) data set rather than a multi–year cross–section. Panel data has advantages over cross–sectional data, as the analysis can be used to exploit both inter–individual differences and intra–individual dynamics, and as it allows for more accurate inference of model parameters, controlling the impact of omitted variables, uncovering dynamic relationships and providing micro foundations for aggregate data analysis [25]. Each observation in the data set included information on the age and county of residence of the patient in that year and the total number of PHC, outpatient, and inpatient contacts for the selected ACSCs. Multimorbidity was defined as the number of different diagnoses (of the seven ACSCs analysed) for the patient recorded in primary, outpatient or inpatient care in the given year.

Trends in patient–level utilisation patterns for primary, outpatient, and inpatient care were examined by patient age, gender, specific conditions and multimorbidity status (one, two, three, and four or more recorded conditions). While we only present summary statistics for the earliest and latest year of data for each stratum, we tested the statistical significance of yearly trends using basic linear regression models, which are reported in *P*–values in text and in tables.

For patient level multivariate analyses, the outcome of interest is the probability of an inpatient admission in a given year, for patients that had at least one health care contact for any of the seven ACSCs. Given the panel and nested structure of the data set (patients are ‘clustered’ in counties), the appropriate model of analysis is a multi–level regression, with county level random effects, and robust standard errors [26], to control for intra–cluster correlation. The model specification is: 

where H(.) is the logistic cumulative distribution function, mapping linear predictor to the probability of an admission (*y_i,j,t_* = 1) with H(v) = exp(v)/(1+exp(v)). The regression coefficient *y* provides the estimate for the yearly trend in the probability of an inpatient admission, adjusted for patient level explanatory variables (*x_i,t_*: age, gender, and number of conditions) and access to care as measured by county level supply side variables (*z_j,t_*: number of hospitals, beds, PHC centres and doctors in PHC per 1000 population, nurse to doctor ratio in PHC, and log average disposable income in the county).

Using patient level data, we also explored the association between PHC visits and rate of inpatient admissions by patient age, gender and multimorbidity status for 2005 and 2012. For each subgroup of patients, we report the rate of inpatient admissions for patients who had no PHC visits for the seven ACSCs and those who had at least one PHC visit. We provide the logistic regression estimates of the crude and adjusted odds of inpatient admission, for patients who had at least one PHC visit relative to those who did not in the same year. All regression results have robust standard errors controlling for county level clusters.

## RESULTS

Between 2005 and 2012, 552 822 unique patients accessed health services for at least one of the seven selected ACSCs. The total number of patient years covered by the study was 2 257 347. The number of patients accessing health services per year ranged from approximately 260 000 to 300 000, corresponding to 22.0–27.0% of Estonian population aged 15 years and older. Around 63.2% of the patients using health services was female, and the mean age of the patients was 63.3 years.

Approximately 76.7% of the patients presented to services with only one ACSC in a given year, 19.2% had two ACSCs and 4.1% had three or more ACSCs. Hypertension was the most frequent condition in PHC contacts for ACSCs in a given year, with 75.4% of all patients having at least one PHC contact due to hypertension, followed by IHD and diabetes (14.3% and 12.2%, respectively).

The total number of contacts (PHC, outpatient and inpatient) for the seven ACSCs increased from 936 365 in 2005 to 1 247 522 in 2012. The majority of health care contacts occurred in PHC and at outpatient clinics. In 2005, 77.8% of the total contacts were in PHC, 20.0% in outpatients and 2.2% in inpatients. By 2012, the relative proportion for PHC visits had increased to 81.1% of total contacts, while outpatient visits declined to 17.5%, and inpatient admissions fell to 1.4% (**Table 1**).

**Table 1:** Total contacts (consultations and hospitalisations) for seven selected conditions (2005–2012) and distribution by primary, outpatient and inpatient care.

	2005	2006	2007	2008	2009		2010	2011	2012
Total contacts*	936 365	973 896	1 031 422	1 181 308	1 137 760		1 179 355	1 202 887	1 247 522
Age–standardised rate†	91 888	95 221	100 366	114 349	109 454		112 718	114 157	117 516
**Percentage of contacts by service tier:**									
PHC	77.8	77.1	77.9	78.6		79.1	80.4	80.2	81.1
Outpatient	20.0	20.6	20.0	19.5		19.2	18.0	18.3	17.5
Inpatient	2.2	2.2	2.1	1.8		1.7	1.6	1.5	1.4

PHC – Primary Health Care

*Includes all consultations and hospitalisations.

†Per 100 000 population.

**Figure 1:**
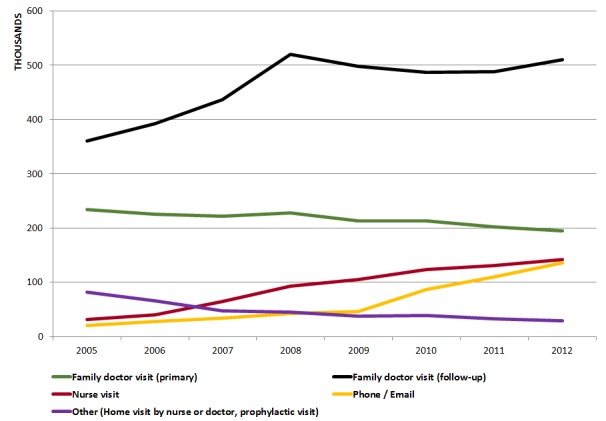
Total number of consultations in primary health care by type.

**Figure 2:**
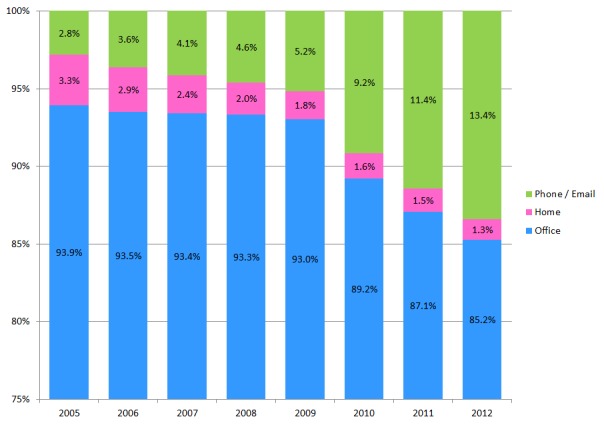
Proportion of primary health care consultations by type.

PHC consultations for ACSCs rose by 38.8%, from 728 885 in 2005 to 1 011 906 in 2012. More than 90% of consultations took place in family doctors’ offices, with the proportion decreasing from 93.9% to 85.2% of PHC consultations during the study period. Home visits as a percentage of all PHC contacts also declined (3.3% to 1.3%). There was a six–fold increase in the use of phone consultations in PHC from 20 000 calls in 2005 to 135 000 in 2012 (2.8% to 13.4%), and email consultations, which have been recorded since 2010, rose to 907 in 2012 (**Figure 1** and **Figure 2**).

There were small differences in the type of visit by patient gender, age group and condition. In 2012, patients who were aged greater than 75 years were more likely to have home visits (3.2%) or phone consultations (15.1%) than patients in other age groups, whereas younger patients were more likely to have email consultations. Patients with a diagnosis of heart failure were most likely to have a home visit (5.0%), whereas patients with asthma and depression used phone/email consultations more frequently than patients with other conditions (14.2% and 14.0%, respectively).

Between 2005 and 2012, new consultations decreased by 16.6% (from 233 044 to 194 293) and follow–up consultations increased by 41.5% (from 360 454 to 510 120). The proportion of follow–up consultations among episodes of care in family doctors’ offices increased from 55.3% to 70.8%.

While the number of family physicians per capita stayed relatively constant during the study period, the number of full–time equivalent (FTE) nurses in family doctors’ offices increased by 26.7% from 0.49 to 0.63 per 1000 population. Nurse consultations increased four–fold, from 34 253 to 145 348. Patients with hypertension, diabetes and heart failure were more likely to be consulted by a nurse as compared with patients with other ACSCs.

Among patients that accessed PHC for any of the ACSCs, the average number of visits per patient was 2.8 in 2005, increasing to 3.4 visits in 2012. Average number of outpatient visits remained stable at 0.72 per patient, however. A decreasing number of patients had at least one inpatient admission during the year: 16 541 patients (6.4%) in 2005 and 13 674 (4.6%) in 2012. The average number of inpatient admissions per patient decreased from 0.079 to 0.056 between 2005 and 2012. Females had higher number of PHC visits on average, while males had higher outpatient consultations and inpatient admissions in both 2005 and 2012 (Figure S1 in **Online Supplementary Document(Online Supplementary Document)**).

**Figure 3:**
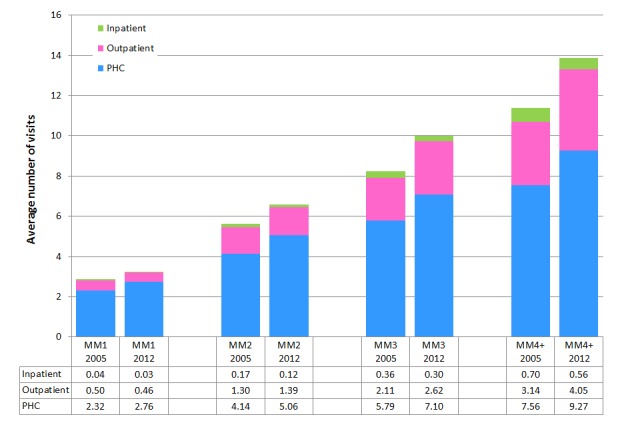
Number of visits in primary, outpatient and inpatient care, by multimorbidity group, 2005 and 2012. MM1 – 1 multi–morbidity, MM2 – 2 multi–morbidities, MM3 – 3 multi–morbidities, MM4+ – 4 or more multi–morbidities.

**Figure 4:**
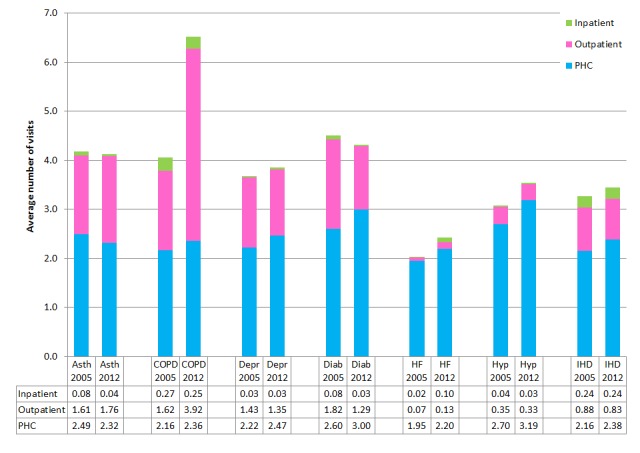
Average number of visits in primary, outpatient and inpatient care, by condition, 2005 and 2012. Asth – asthma; COPD – chronic obstructive pulmonary disease; Depr – depression; Diab – diabetes; HF – heart failure; Hyp – hypertension; IHD – ischemic heart disease.

The average number of visits to PHC and hospitals rose with increasing age and multi–morbidity, with the highest inpatient admission rate observed for patients that had four or more ACS conditions in a given year, and in particular for those aged 55 and above. (**Figure 3**, **Table 2**, and Figure S2 in **Online Supplementary Document(Online Supplementary Document)**). Healthcare utilisation varied by condition: patients that had at least one health contact in a given year with a primary diagnosis of diabetes or hypertension utilised PHC services more frequently than patients with other diagnoses. Average number of outpatient visits was higher for patients with diabetes, COPD, asthma and depression, and rose significantly for patients with COPD in 2012, whereas patients with IHD or COPD had more frequent inpatient admissions (**Figure 4**).

**Table 2:** Average number of inpatient admissions, by age and multimorbidity (MM) group, 2005 and 2012

		MM1		MM2		MM3		MM4+	
**Age, year**	**Number of patients**	%*	**Mean**	%*	**Mean**	%*	**Mean**	%*	**Mean**
15–44, 2005	32 603	94.3	0.023	5.4	0.082	0.3	0.162	0.0	0.429
15–44, 2012	34 120	93.8	0.019	5.9	0.057	0.4	0.108	0.0	0.417
45–54, 2005	36 624	84.8	0.034	13.6	0.148	1.4	0.312	0.1	0.667
45–54, 2012	39 114	85.6	0.018	13.0	0.082	1.4	0.175	0.1	0.571
55–64, 2005	55 050	78.3	0.038	18.5	0.179	2.9	0.365	0.4	0.724
55–64, 2012	69 122	78.4	0.022	18.3	0.102	2.9	0.266	0.4	0.627
65–74, 2005	70 603	71.8	0.045	23.2	0.177	4.4	0.366	0.7	0.712
65–74, 2012	73 284	73.4	0.028	21.6	0.125	4.2	0.303	0.7	0.553
75+, 2005	63 335	66.9	0.049	26.4	0.175	5.9	0.367	0.9	0.690
75+, 2012	83 799	68.6	0.038	24.8	0.143	5.6	0.321	1.0	0.550

MM1 – 1 multi–morbidity; MM2 – 2 multi–morbidities; MM3 – 3 multi–morbidities; MM4+ – 4 or more multi–morbidities

*Percentages shown are of total admissions for that age–group and year.

Age standardised rates for total health service contacts were 91.9 per 1000 population in 2005 and 117.5 per 1000 population in 2012. PHC utilisation rate was highest (by a significant proportion) for patients with hypertension, followed by diabetes in 2005 and IHD in 2012 (Figure S3 in **Online Supplementary Document(Online Supplementary Document)**). Rates of outpatient visits were also highest for patients with hypertension and diabetes (Figure S4 in **Online Supplementary Document(Online Supplementary Document)**). Inpatient admission rates were highest for IHD and hypertension in both years (Figure S5 in **Online Supplementary Document(Online Supplementary Document)**).

**Figure 5:**
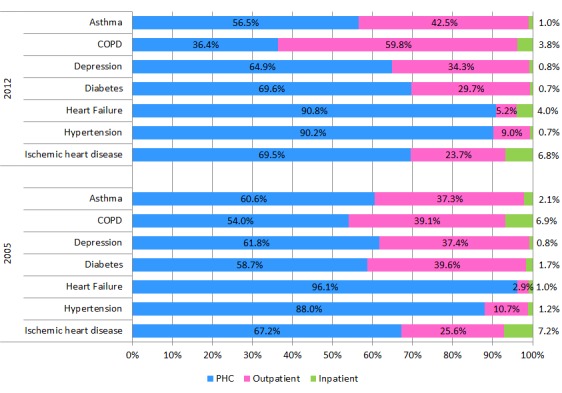
Distribution of utilization of total consultations and hospital admissions for seven selected conditions between primary health care (PHC), outpatient, and inpatient services (2005 and 2012).

The utilisation levels between the three service elements for each ACSC vary (**Figure 5**). During the study period, of the seven ACSCs, more than 85% of service utilisation for heart failure and hypertension was in PHC; followed by IHD, depression, and diabetes (63–68%), asthma and COPD (57% and 41% respectively).

Across all gender, age and multimorbidity groups, average number of PHC visits increased between 2005 and 2012 (*P* ≤ 0.01) and inpatient admissions decreased (*P* ≤ 0.01) (**Table 3**). Patterns of outpatient visits varied by groups: outpatient utilisation for patients aged 75+ (*P* = 0.001) and patients with three ACSCs rose (*P* = 0.03), but declined for those aged for 45–54 (*P* = 0.01) and for patients with one ACSC only (*P* = 0.002).

**Table 3:** Annual trends in utilisation measures by selected patient characteristics, univariate linear regression model

	PHC visits	Outpatient visits	Inpatient visits
**Gender:**			
Male	Increase (*P* = 0.001)	No change (*P* = 0.57)	Decrease (*P* < 0.001)
Female	Increase (*P* = 0.001)	No change (*P* = 0.41)	Decrease (*P* < 0.001)
**Age:**			
15–44	Increase (*P* = 0.001)	No change (*P* = 0.48)	Decrease (*P* = 0.004)
45–54	Increase (*P* = 0.01)	Decrease (*P* = 0.01)	Decrease (*P* < 0.001)
55–64	Increase (*P* = 0.004)	No change (*P* = 0.56)	Decrease (*P* < 0.001)
65–74	Increase (*P* = 0.001)	No change (*P* = 0.46)	Decrease (*P* < 0.001)
75+	Increase (*P* < 0.001)	Increase (*P* = 0.004)	Decrease (*P* = 0.002)
**Multimorbidity:**			
1	Increase (*P* = 0.001)	Decrease (*P* = 0.002)	Decrease (*P* < 0.001)
2	Increase (*P* < 0.001)	No change (*P* = 0.18)	Decrease (*P* < 0.001)
3	Increase (*P* < 0.001)	Increase (*P* = 0.03)	Decrease (*P* = 0.002)
4+	Increase (*P* < 0.001)	No change (*P* = 0.29)	Decrease (*P* < 0.001)
**Condition–specific, mean visits per patient:**			
Asthma	No change (*P* = 0.06)	No change (*P* = 0.55)	Decrease (*P* < 0.001)
COPD	Increase (*P* = 0.01)	Increase (*P* = 0.05)	Decrease (*P* = 0.02)
Depression	Increase (*P* = 0.005)	No change (*P* = 0.74)	No change (*P* = 0.12)
Diabetes	Increase (*P* = 0.004)	Decrease (*P* < 0.001)	Decrease (*P* < 0.001)
Heart failure	Increase (*P* = 0.001)	Increase (*P* = 0.001)	Increase (*P* < 0.001)
Hypertension	Increase (*P* = 0.001)	Decrease (*P* = 0.03)	Decrease (*P* = 0.001)
IHD	Increase (*P* = 0.02)	No change (*P* = 0.78)	No change (*P* = 0.41)
**Condition–specific, utilisation rates per 100 000 population:**			
Asthma	Increase (*P* < 0.001)	Increase (*P* = 0.002)	Decrease (*P* < 0.001)
COPD	No change (*P* = 0.29)	No change (*P* = 0.06)	Decrease (*P* = 0.01)
Depression	No change (*P* = 0.15)	No change (*P* = 0.20)	No change (*P* = 0.11)
Diabetes	Increase (*P* < 0.001)	No change (*P* = 0.07)	Decrease (*P* = 0.01)
Heart failure	Decrease (*P* < 0.001)	Increase (*P* = 0.003)	Increase (*P* < 0.001)
Hypertension	Increase (*P* < 0.001)	Increase (*P* = 0.02)	Decrease (*P* = 0.04)
IHD	Decrease (*P* < 0.001)	Decrease (*P* < 0.001)	Decrease (*P* < 0.001)

PHC – primary health care; COPD – chronic obstructive pulmonary disease; IHD – ischaemic heart disease

Service utilisation trends for average number of condition–specific contacts for patients with a primary diagnosis of a condition in a given year also varied. Average number of PHC visits increased for all ACSCs except asthma. Outpatient visits increased for heart failure and COPD, yet declined for diabetes and hypertension. The number of inpatient admissions decreased for asthma, COPD, diabetes and hypertension, but increased for heart failure.

The age–standardised rate for total health service contact increased by 27.9% during the study period from 91 888 to 117 516. For PHC and outpatient services, total utilisation for ACSCs increased by 32.0% (from 72 491 to 95 720) and 16.4% (from 17 347 to 20 193) respectively, whereas age–standardised inpatient admissions rates declined by 21.8% from 2051 to 1604. Yearly trends in PHC utilisation rates were significant for all ACSCs except COPD and depression, with PHC utilisation falling for IHD and heart failure (*P* < 0.001) and rising for asthma, diabetes and hypertension (*P* < 0.001). Outpatient utilisation rates were comparable during the study period for COPD, depression and diabetes, decreased for IHD and increased for all remaining ACSCs. Inpatient admission rates increased for heart failure and decreased for asthma, COPD, diabetes, hypertension and IHD. The significance of yearly trends in age–standardised inpatient admission rates remained in multivariate, county–year panel regressions with county random effects, adjusting for demographics and disease burden (% of patients over 65 years of age, % of patients with three or more ACSC), supply side variables (number of hospitals, beds, PHC centres and doctors in PHC per 1000 population, nurse to doctor ratio in PHC) and socioeconomic factors (disposable income, employment rate) (Table S1 in **Online Supplementary Document(Online Supplementary Document)**).

For diabetes, depression, IHD and hypertension there is a shift towards greater utilisation in PHC (**Figure 5**). For diabetes PHC utilisation increased from 58.7% of total episodes in 2005 to 69.6% in 2012, whereas outpatient and inpatient episodes fell from 39.6% to 29.7% and 1.7% to 0.7%, respectively. There was a similar, albeit smaller, shift in the proportional utilisation of PHC for depression, IHD and hypertension. For depression, PHC utilisation rose from 61.8% to 64.9%, IHD from 67.2% to 69.5% and hypertension from 88.0% to 90.2% of total, with concomitant reductions in the share of utilisation in outpatient and inpatient episodes.

Conversely, COPD utilisation in PHC as a proportion of total episodes decreased from 54.0% in 2005 to 36.4% in 2012, inpatient episodes declined from 6.9% to 3.8% whilst outpatient episodes grew from 39.1% to 59.8% of total. For heart failure, utilisation of PHC as a proportion of total fell from 96.1% to 90.8% while inpatient and outpatient proportions rose from 1.0% to 4.0% and 2.9% to 5.2%, respectively. For asthma, share of outpatient episodes increased, while that for PHC and inpatient admissions fell.

Using patient–year level data, we applied the multivariate regression model to examine the probability of inpatient admissions for patients that accessed health services in a given year for any condition, controlling for patient characteristics and availability of services in the county of residence (**Table 4**). The overall time trends were significant, with the probability of inpatient admissions declining in the study period (odds ratio OR 0.932, 95% CI 0.927, 0.936).

**Table 4:** Multivariate regression model of inpatient admissions (multi–level logistic regression with county random effects and robust standard errors, n = 2 257 347 patient–year observations)

	OR	L 95% CI	U 95%CI	*P*–value
**0.932**	**0.927**	**0.936**	**<0.001**
**Patient characteristics:**				
Female	0.603	0.596	0.611	<0.001
Age group:				
15–44	1.000	–	–	–
45–54	1.344	1.301	1.388	<0.001
55–64	1.622	1.575	1.671	<0.001
65–74	1.974	1.918	2.032	<0.001
75+	2.413	2.345	2.484	<0.001
**Multimorbidity:**				
1	1.000	–	–	–
2	4.317	4.261	4.374	<0.001
3	10.049	9.860	10.241	<0.001
4+	20.065	19.320	20.839	<0.001
**Regional characteristics:**				
Number of hospitals	0.179	0.026	1.223	0.079
Number of beds	1.011	0.985	1.037	0.424
FTE doctors in FM	1.056	0.855	1.305	0.610
Ratio of nurses/doctors in FM	0.955	0.858	1.063	0.402
Disposable income (log)	1.224	1.154	1.297	<0.001
**Random effects (county)**				
*var(_cons): 0.255 (se 0.051)*				

OR – odds ratio, L 95% CI – lower 95% confidence interval, U 95% CI – upper 95% confidence interval, FTE – full–time equivalent; FM – family medicine

The odds of inpatient admission was higher for males, and increased with age and multimorbidity: patients over 65 years of age were two times more likely to have an inpatient admission as compared with patients 15–44 years of age (Age 65–74 OR 1.97, 95% CI 1.91, 2.03; Age 75+ OR 2.41, 95% CI 2.35, 2.48). Patients with four or more ACSCs were 20 times more likely to have an inpatient admission than patients with a single ACSC (95% CI 19.3, 20.8). In counties with higher average income, the likelihood of inpatient admissions was higher. Other county level variables, such as hospitals, beds and number of physicians per capita were not statistically significant.

In 2005, among the patients who had any health contact with the seven ACSCs, 8.2% did not have any PHC contact (had only outpatient or inpatient contact) for these conditions, declining to 6.7% in 2012. The proportion of patients who did not have any PHC contact was higher for males and decreased by age and multimorbidity groups. Those who did not have any PHC contact were more likely to have an inpatient admission than patients with at least one PHC contact (0.121 vs 0.059; adjusted OR 0.20, 95% CI 0.15, 0.29). This association held for 2012, as well as for all patient gender, age and multimorbidity groups (**Table 5**).

**Table 5:** Inpatient admissions, by primary care attendance in the same year

			Inpatient admissions rate		Odds of inpatient admissions with at least one PHC contact*	
	**Number of patients**	**% No. PHC contact**	**No PHC contact**	**1+ PHC contact**	**Crude OR (95% CI)**	**Adjusted OR† (95% CI)**
ALL 2005	258 215	8.2	0.121	0.059	0.45 (0.36, 0.57)	0.20 (0.15, 0.25)
ALL 2012	299 439	6.7	0.107	0.041	0.36 (0.31, 0.42)	0.13 (0.11, 0.16)
**By gender:**						
Males 2005	82 280	10.0	0.140	0.074	0.49 (0.40, 0.61)	0.22 (0.17, 0.27)
Males 2012	112 930	7.8	0.128	0.050	0.36 (0.31, 0.42)	0.13 (0.11, 0.17)
Females 2005	163 810	7.3	0.107	0.051	0.45 (0.35, 0.57)	0.18 (0.13, 0.23)
Females 2012	186 509	6.1	0.091	0.036	0.37 (0.31, 0.44)	0.13 (0.10, 0.16)
**By age group:**						
15–44, 2005	32 603	22.3	0.047	0.018	0.38 (0.30, 0.48)	0.31 (0.24, 0.40)
15–44, 2012	34 120	20.2	0.042	0.012	0.28 (0.24, 0.34)	0.22 (0.18, 0.28)
45–54, 2005	36 624	11.1	0.084	0.041	0.46 (0.40, 0.54)	0.32 (0.26, 0.39)
45–54, 2012	39 114	9.0	0.068	0.021	0.29 (0.25, 0.34)	0.18 (0.15, 0.21)
55–64, 2005	55 050	8.0	0.113	0.056	0.46 (0.36, 0.59)	0.28 (0.21, 0.38)
55–64, 2012	69 122	6.1	0.092	0.034	0.34 (0.31, 0.39)	0.19 (0.16, 0.22)
65–74, 2005	70 603	5.1	0.185	0.068	0.32 (0.25, 0.43)	0.19 (0.13, 0.26)
65–74, 2012	73 284	4.2	0.159	0.046	0.26 (0.20, 0.33)	0.13 (0.09, 0.19)
75 +, 2005	63 335	3.1	0.378	0.078	0.14 (0.10, 0.19)	0.06 (0.04, 0.09)
75 +, 2012	83 799	2.9	0.307	0.061	0.15 (0.12, 0.19)	0.07 (0.05, 0.09)
**By multimorbidity:**						
MM1, 2005	197 950	10.1	0.114	0.026	0.21 (0.16, 0.26)	0.16 (0.13, 0.20)
MM1, 2012	230 954	8.3	0.099	0.016	0.15 (0.12, 0.17)	0.11 (0.09, 0.13)
MM2, 2005	49 976	2.4	0.237	0.133	0.50 (0.40, 0.61)	0.47 (0.40, 0.56)
MM2, 2012	56 367	1.8	0.229	0.097	0.36 (0.29, 0.44)	0.32 (0.27, 0.39)
MM3, 2005	8986	0.8	0.306	0.262	0.81 (0.58, 1.11)	0.78 (0.56, 1.07)
MM3, 2012	10 399	0.8	0.481	0.212	0.29 (0.21, 0.40)	0.28 (0.20, 0.39)
MM4 +, 2005	1303	0.2	0.667	0.448	N/A‡	N/A‡
MM4 +, 2012	1719	0.1	<0.001	0.370	N/A‡	N/A‡

PHC – Primary Health Care, MM1 – 1 multi–morbidity, MM2 – 2 multi–morbidities, MM3 – 3 multi–morbidities, MM4+ – 4 or more multi–morbidities, OR – odds ratio

*ORs derived from logistic regression model for the subgroup, with any inpatient admissions as the dependent variable, and any PHC attendance in the same year as the explanatory variable. Reference group: patients with no PHC contact (OR = 1).

†Adjusted ORs also control for age groups and multimorbidity for subgroups by gender; gender and multimorbidity for age subgroups; and gender and age for multimorbidity subgroups.

‡There are less than 5 cases in the subgroup, regression results not available.

## DISCUSSION

The results indicate increasing overall utilisation of health care services in 2005–2012 for the seven ACSCs analysed. For these seven ACSCs, there was increased utilisation of PHC, with a concomitant fall in inpatient admissions. These trends were observed for all patients, but the utilisation rates rose with age and particularly, with multimorbidity.

The observed trends varied by condition. For asthma, diabetes and hypertension, PHC utilisation as a proportion of total number of contacts rose. Inpatient admissions fell significantly for all conditions except for heart failure. The results point to a shift in care towards PHC–particularly for diabetes and hypertension.

Across the period of analysis, the nature of PHC provision changed: there was an increase in follow–up consultations in PHC as the predominant share of visits, as well as an increase in the number and proportion of consultations with nurses and those using phone and email.

The fall in referral to inpatient admission and outpatient departments has been coupled with increasing numbers of appointments for follow–up consultations in PHC, indicating increased management of these conditions in PHC setting.

High levels of admissions for ACSCs frequently indicates inadequate co–ordination between elements of the health system, and is an indicator of poor overall quality of PHC [27] – particularly for continuity of care [28,29]. There is evidence that better quality PHC, through attainment of financial quality indicators, leads to reductions in hospitalisations for certain ACSCs [30] including diabetes [31–33], COPD [34], but not heart disease [35]. There is also evidence that case management of only high–risk patients with chronic illness at the PHC level may not lead to reduced secondary care admissions in all contexts [36]. Increased access to PHC could potentially decrease emergency admissions [37], and improvements in PHC quality suggest potential cost savings through reduced emergency admissions and outpatient visits [38]. Reductions in admissions of ACSCs in Estonia may be indicative of improvements in the overall quality and continuity of PHC for all patients.

In Estonia, the observed changes in utilisation have been mediated mainly through supply–side changes, such as the introduction of family medicine, and nurses working in family medicine centres, alongside financial changes introduced by the EHIF. Specialist outpatient care was prioritised over inpatient care, with the introduction of a quality bonus system [39,40] and clinical guidelines in PHC, and in response to the 2009 financial crises [41], containment of specialist care growth and reduction of inpatient care [42,43]. Demand side interventions included a visit fee for specialist care while PHC was kept free at the point of service delivery [3].

In spite of significant shifts, a recent study has noted challenges faced by Estonian health system, with high levels of specialist care and long hospital stays. Financial incentives to increase hospital care and lack of capacity to transfer care out of specialist settings were noted as the main reasons promoting hospital use, but inadequacies in PHC were also identified as a contributing factor to high levels of hospital and specialists use [44].

Worldwide, NCDs and multi–morbidity are rising rapidly [45]. Estonia is no exception. Patients with multi–morbidity have higher health system utilisation [16,46]. In Estonia, multimorbidity prevalence increases with age [47], as risk factors and chronic disease accumulate over the years [48]. In Estonia multimorbidity was associated with significantly high levels of outpatient and inpatient utilisation [49]. Patients with four or more ACSCs were 20 times more likely to have an inpatient admission than patients with a single ACSC. In our data, patients with multimorbidity accounted for 59.4% of PHC visits [50].

Multimorbidity is a major challenge to health systems, and prevailing approaches that focus on a single disease lead to fragmented care [51]. PHC has a central role in managing NCDs and multimorbidity [47,52], which require, effective co–ordination and care–continuity across all levels of care [53]. Integrated care that enables interventions across multiple levels of the health system with “connectivity, alignment and collaboration” [54] can help effective management of multimorbidity [55].

Our analysis shows that patients were significantly less likely to have an inpatient admission if they had any PHC visit in the given year, suggesting a protective effect of PHC consultations.

We use an observational study design and do not set out to demonstrate causality on the impact of health system reforms on health service utilisation, but where possible we use a comprehensive data set with robust methods to control for confounders to produce plausible evidence [56]. Although there is potential from error from data quality, the nature of the nationwide comprehensive data set – based on invoicing with built in controls and record validation checks – ensures our findings are reliable.

The comprehensive and detailed data used in this analysis has enabled robust analysis to provide deeper understanding of health care utilisation trends in Estonia. As all patient consultations and admissions are recorded, a complete analysis of the country was possible. The EHIF database has in–built quality checks and is subject to retrospective quality analysis to ensure reliable data. Each inpatient admission and outpatient visit is linked to reimbursement so data undergo scrutiny. In PHC–paid by a mixture of capitation, fee–for–service and performance related pay– physicians report activities to EHIF, and the data are checked to confirm reporting. The nationwide size and completeness of the data set, and regular quality checks mean potential reporting errors should be small.

The seven ACSCs used to examine changes in health care utilisation over time – all of which are high prevalence conditions that are likely to be sensitive to changes in service availability and quality, and represent a subset of all the conditions encountered in Estonia. Whilst we demonstrate changes in these seven ACSCs, there is the potential that concurrently changes in other conditions may negate the positive impacts concluded in this study. While this is a potential, these seven conditions represent a large burden for Estonia and are important in their own right as major NCDs that require effective management and coordination, and are good indicators of PHC quality.

Our analysis of multi–morbidity drew on only the seven ACSCs we had data for, whilst previous studies have recommended examining greater than 12 chronic diseases to report two or more concurrent diseases [57]. However, we have not attempted to report a prevalence of multimorbidity, but merely used the measure as a subgroup analysis, therefore we consider these methods adequate for this purpose. Furthermore, our analysis included depression which is likely to be particularly important in multimorbidity management and outcomes [21].

Notwithstanding limitations, the study provides compelling evidence of the positive effects of family–medicine centred health system reforms on expanding PHC utilisation and reducing hospital inpatient admissions for key NCDs–important for many countries globally that have committed to providing UHC and have to efficiently manage the rising burden of chronic illness.
