# Supplementary material for: Shifting chronic disease management from hospitals to primary care in Estonian health system: analysis of national panel data
Source: J Glob Health. 2016 Aug 19;6(2):020701. doi: 10.7189/jogh.06.020701 (PMC5017034; doi:10.7189/jogh.06.020701)
Supplement: Online Supplementary Document [file jogh-06-020701-s001.pdf]

Online Supplementary Document

Atun et al. Shifting chronic disease management from hospitals to primary care in Estonian health system: econometric analysis of national panel data

JoGH 2016;6:020701

Figure S1. Number of visits in primary, outpatient and inpatient care, all and by gender, 2005 and 2012

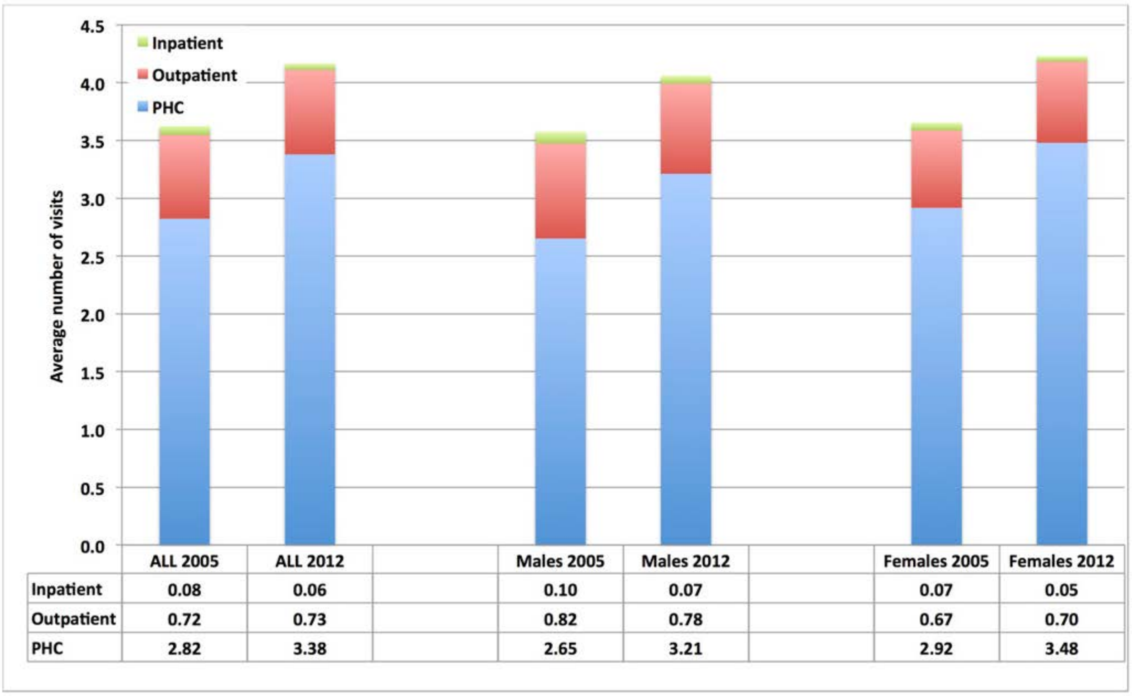

	ALL 2005	ALL 2012		Males 2005	Males 2012		Females 2005	Females 2012
Inpatient	0.08	0.06		0.10	0.07		0.07	0.05
Outpatient	0.72	0.73		0.82	0.78		0.67	0.70
PHC	2.82	3.38		2.65	3.21		2.92	3.48

Figure S2. Number of visits in primary, outpatient and inpatient care, by age group, 2005 and 2012

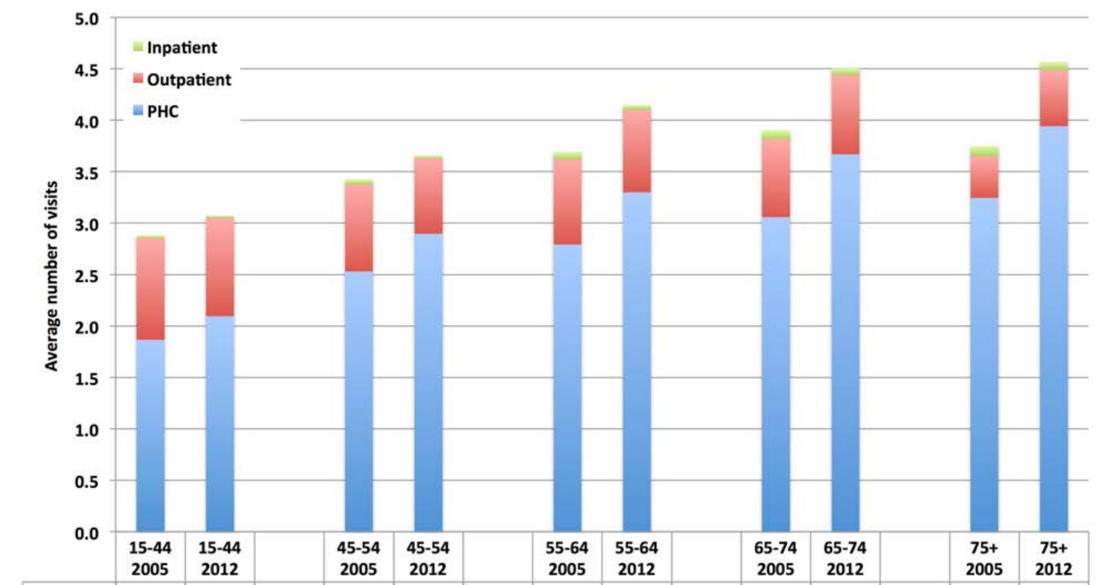

	15-44 2005	15-44 2012		45-54 2005	45-54 2012		55-64 2005	55-64 2012		65-74 2005	65-74 2012		75+ 2005	75+ 2012
Inpatient	0.03	0.02		0.05	0.03		0.08	0.05		0.09	0.06		0.11	0.08
Outpatient	0.99	0.96		0.84	0.73		0.83	0.80		0.75	0.77		0.40	0.54
PHC	1.87	2.09		2.53	2.90		2.79	3.30		3.06	3.67		3.25	3.94

Figure S3. Age-standardised consultation (per 100,000 population) in primary health care for selected conditions (2005 and 2012)

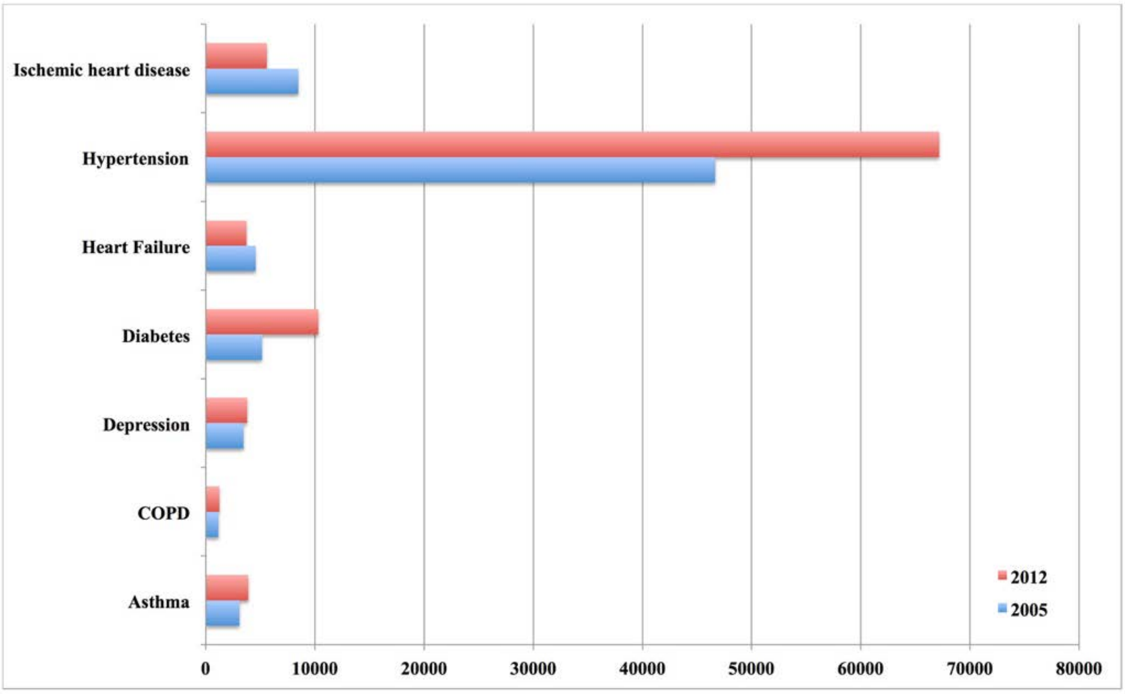

Figure S4. Age-standardised consultation rates (per 100,000 population) in outpatients for selected conditions (2005 and 2012)

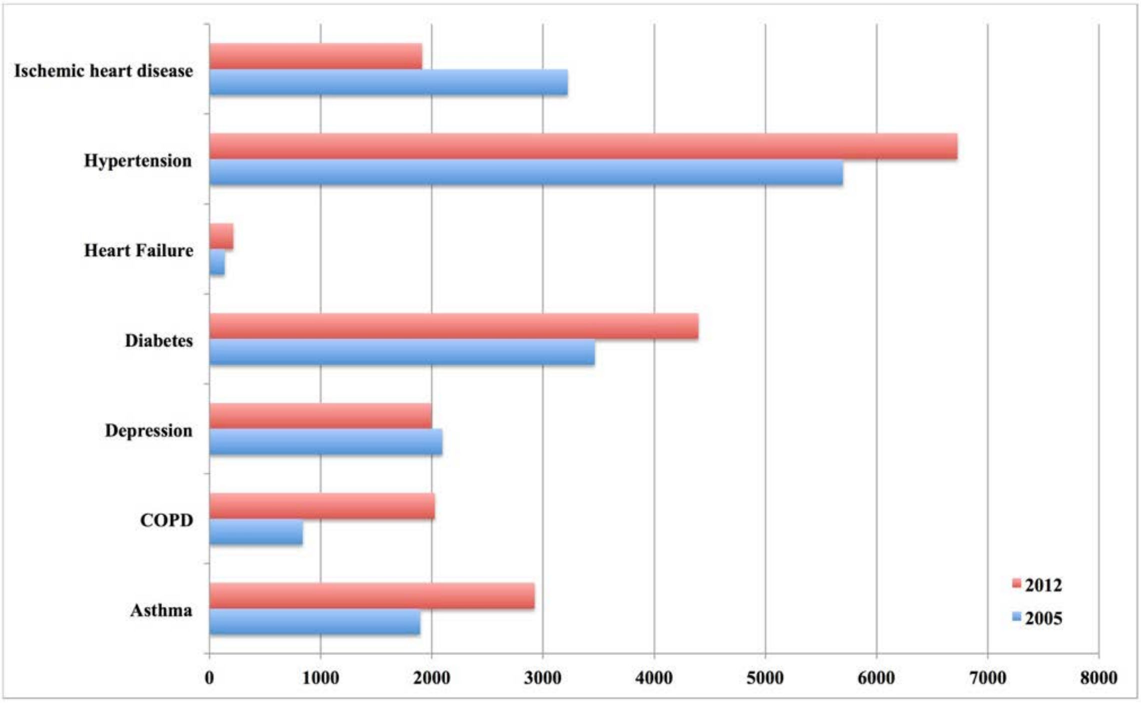

Figure S5. Age-standardised admission (inpatient) rates (per 100,000 population) for selected conditions (2005 and 2012)

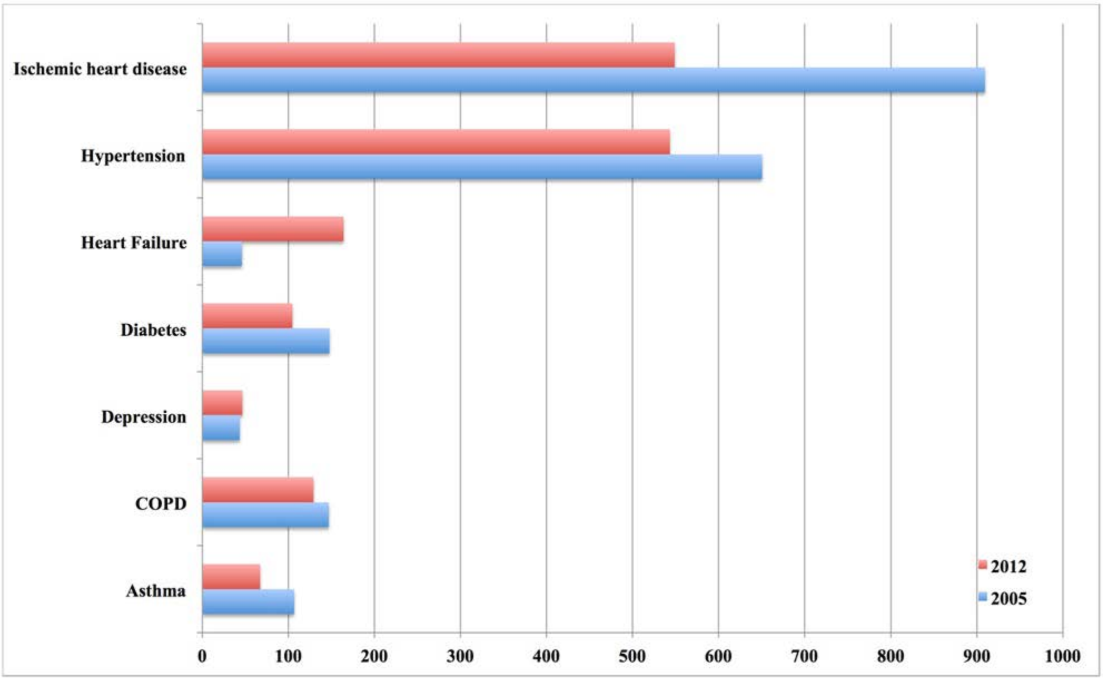

Table S1. Multivariate regression models of age-standardised inpatient admission rates, by condition. (Linear regression with county level random effects, n=120 county-year observations)

	Total	Asthma	COPD	Depression	Diabetes	Heart Failure	Hypertension	IHD
**Year**	**-**	**-**	**-**	**ns**	**-**	**+**	**-**	**-**
% of patients aged 65+	ns	ns	ns	ns	ns	ns	ns	ns
% of patients with MM3+	ns	-	+	ns	ns	ns	ns	ns
Number of hospitals	+	ns	ns	ns	ns	ns	+	ns
Number of hospital beds	ns	ns	ns	ns	ns	ns	ns	ns
Doctors in FM (FTE)	ns	ns	ns	ns	ns	-	ns	ns
Nurse / Doctor ratio in FM	ns	ns	+	ns	-	ns	-	+
Employment rate	ns	ns	ns	ns	ns	ns	ns	ns
Disposable income (log)	ns	ns	ns	ns	ns	ns	+	ns
Population (log)	ns	ns	ns	ns	ns	ns	-	ns

ns: $p>0.05$; + positively associated with inpatient admissions ($p<0.05$); - negatively associated with inpatient admissions ($p<0.05$)
COPD- Chronic Obstructive Pulmonary Diseease; IHD – Ischaemic Heart Disease; MM3+ - 3 or more multi-morbidities; FM – Family Medicine; FTE – Full-time Equivalent;
